# Comparative Analyses of Five Complete Chloroplast Genomes from the Genus *Pterocarpus* (Fabacaeae)

**DOI:** 10.3390/ijms21113758

**Published:** 2020-05-26

**Authors:** Zhou Hong, Zhiqiang Wu, Kunkun Zhao, Zengjiang Yang, Ningnan Zhang, Junyu Guo, Luke R. Tembrock, Daping Xu

**Affiliations:** 1 State Key Laboratory of Tree Genetics and Breeding, Research Institute of Tropical Forestry, Chinese Academy of Forestry, Guangzhou 510520, China; hzhou1981@caf.ac.cn (Z.H.); zhaokunkun@caf.ac.cn (K.Z.); yzengjiang@caf.ac.cn(Z.Y.); ningnanzhang@caf.ac.cn (N.Z.); junyuguo@caf.ac.cn (J.G.); 2 Guangdong Laboratory for Lingnan Modern Agriculture, Genome Analysis Laboratory of the Ministry of Agriculture, Agricultural Genomics Institute at Shenzhen, Chinese Academy of Agricultural Sciences, Shenzhen 518120, China; wuzhiqiang@caas.cn; 3 Department of Agricultural Biology, Colorado State University, Fort Collins, CO 80523, USA; Luke.R.Tembrock@aphis.usda.gov

**Keywords:** Pterocarpus, chloroplast genome, hypervariable regions, microsatellite markers, purifying selection, phylogenetic analysis

## Abstract

*Pterocarpus* is a genus of trees mainly distributed in tropical Asia, Africa, and South America. Some species of *Pterocarpus* are rosewood tree species, having important economic value for timber, and for some species, medicinal value as well. Up to now, information about this genus with regard to the genomic characteristics of the chloroplasts has been limited. Based on a combination of next-generation sequencing (Illumina Hiseq) and long-read sequencing (PacBio), the whole chloroplast genomes (cp genomes) of five species (rosewoods) in Pterocarpus (*Pterocarpus macrocarpus*, *P. santalinus*, *P. indicus*, *P. pedatus*, *P. marsupium*) have been assembled. The cp genomes of five species in *Pterocarpus* have similar structural characteristics, gene content, and sequence to other flowering plants. The cp genomes have a typical four-part structure, containing 110 unique genes (77 protein coding genes, 4 rRNAs, 29 tRNAs). Through comparative genomic analysis, abundant simple sequence repeat (SSR)loci (333–349) were detected in *Pterocarpus*, among which A /T single nucleotide repeats accounted for the highest proportion (72.8–76.4%). In the five cp genomes of *Pterocarpus*, eight hypervariable regions, including trnH-GUG_psbA, trnS-UGA_psbC, accD-psaI, ndhI-exon2_ndhI-exon1, ndhG_ndhi-exon2, rpoC2-exon2, ccsA, and trnfM-CAU, are proposed for use as DNA barcode regions. In the comparison of gene selection pressures (*P. santalinus* as the reference genome), purifying selection was inferred as the primary mode of selection in maintaining important biological functions. Phylogenetic analysis shows that *Pterocarpus* is a monophyletic group. The species *P. tinctorius* is resolved as early diverging in the genus. *Pterocarpus* was resolved as sister to the genus *Tipuana*.

## 1. Introduction

*Pterocarpus* is a genus in the subfamily Papilionoideae of Fabaceae. There are more than 20 tree species in the genus, many of which are highly valued for the quality wood they produce. Species of *Pterocarpus* are mainly distributed in tropical Asia, Africa, and South America [1,2,3]. Some species of this genus are referred to as rosewood (this term also is used to refer to species from the sister genus *Dalbergia*) because of the dark red, high-quality heartwood used for making fine furniture, traditional medicine, and handicrafts such as musical instruments [4]. Because some *Pterocarpus* species are so highly valued for timber and the growth rate is relatively slow, they have been overexploited and, in some cases, may be driven to extinction [5,6]. The loss of genetic diversity caused by human activities increases the risk of species extinction [7]. In 2017, *P. santalinus* (endangered plants) and *P. erinaceus* were listed in the The Convention on International Trade in Endangered Species of Wild Fauna and Flora (CITES) appendices which controls the export of wood from these species because the populations are declining. *Pterocarpus indicus* and *P. angolensis* have been listed as a vulnerable species and a near threatened species by the International Union for Conservation of Nature [8], respectively. Considering the economic value and the loss of genetic diversity of rosewoods species, many countries have begun to restrict export of *Pterocarpus* wood to protect remaining resources [6]. Meanwhile, driven by market demand among other interests [9], it is also common to label wood with similar anatomical features (red color) as a precious wood species such as those from *Pterocarpus* when in fact it might originate from a different genus or even family [9,10]. Owing to the rarity and value of rosewood tree species, research on *Pterocarpus* has mainly focused on the value of developing species for timber production [11,12,13], the proper identification of precious wood [9,10], and the introduction and cultivation for use in timber plantations [14,15,16]. Given the issues of overexploitation and illegal logging associated with high value timber such as rosewood, having molecular markers for species and population identification can be very useful in identifying imposter woods, and tracking illegal logging and exportation [17]. As such, it is vital to have a set of DNA markers to identify the origin and type (species) of high value timber being sold and traded in international markets. In plants, chloroplast genomes have provided numerous such markers for species and population identification.

The chloroplast (cp) is the photosynthetic organelle of plants and algae [18,19]. The cp genome sequence has unique characteristics, such as uniparental inheritance, conserved sequence composition in coding regions, numerous variable sites, and a typical four-part genome structure [20,21,22]. Because of these shared attributes, chloroplast phylogenomics has become a common method to resolve plant phylogenies and evaluate biodiversity [23,24,25]. Research on chloroplast phylogeny and the genomic structural characteristics of *Pterocarpus* is of significance for the protection of biodiversity and for the determination of the appropriate priority protection level of plants [26,27]. *Pterocarpus* is a genus that, due to numerous revisions since the initial naming in 1763, has uncertain affinities in regard to the placement within the Fabaceae [28]. Though some researchers have used molecular methods to explore phylogenetic relationships within the genus and the phylogenetic position of *Pterocarpus* in Papilionoideae [4,29,30], the selected DNA fragments were few and the support values were generally low, resulting in unresolved taxonomic placements. The whole cp genome of three species in *Pterocarpus* had been used to develop DNA barcodes for identifying some rosewood species but did not analyze the structural characteristics of the genome in greater detail [31]. Structural differences among closed species are also valuable in proper species identification. As such, we conducted a detailed comparison of cp genome structural differences to provide additional genomic resources in the study of *Pterocarpus* species.

In this study, we sequenced and analyzed the cp genome of five species (*P. macrocarpus*, *P. santalinus*, *P. indicus*, *P. pedatus*, *P. marsupium*) of *Pterocarpus* (abbreviated throughout the manuscript as M-S-I-P-M2 respectively). In this study, we focused on (1) analyzing the cp genome structural characteristics of *Pterocarpus*, (2) identifying SSR loci to provide resources for later studies in population genetic structure and phylogeography of *Pterocarpus* and its related genera, (3) inferring the phylogenetic relationship of five species in *Pterocarpus* and the phylogenetic position in Fabaceae using the complete cp genome alignments, and (4) identifying hypervariable regions for use as DNA barcodes.

## 2. Results

### 2.1. Genomic Characteristics of Chloroplast

**Figure 1:**
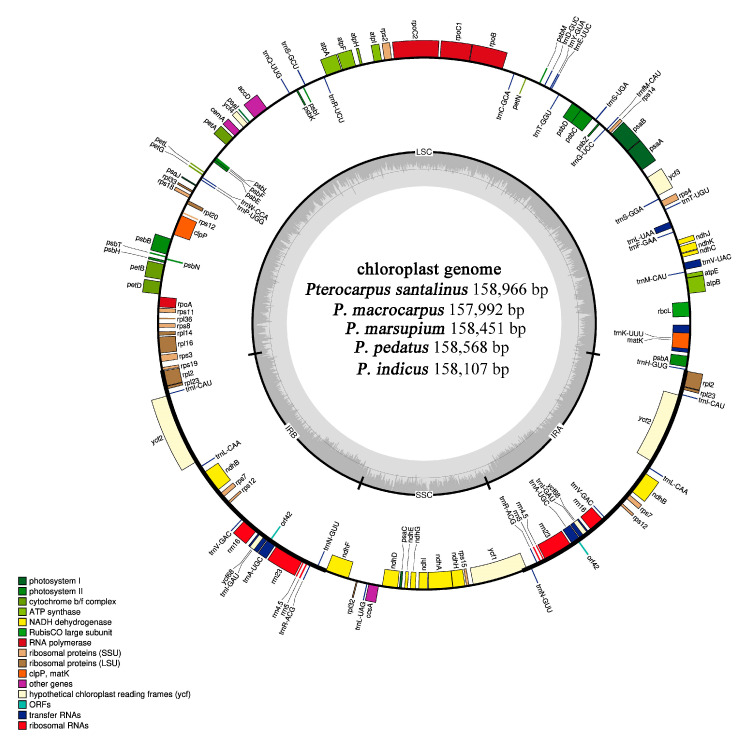
The assembly, size and features of M-S-I-P-M2 cp genomes (*Pterocarpus*). The genes outside the circle are transcribed in the counter clockwise direction, and the genes inside the circle are transcribed in the clockwise direction. Different colors in genes represent different functions. The dark gray area and light gray area of inner circle represent the GC content to AT content of the genome respectively.

Using Illumina Hiseq and PacBio sequencing platforms, we obtained 3666-4403 M (Illumina reads) and 77-335 M PacBio high-quality sequence fragments from five *Pterocarpus* species. After assembly, the length of M-S-I-P-M2 cp genomes were 157,992 bp, 158,966 bp, 158,107 bp, 158,568 bp, and 158,451 bp, respectively. The cp genomes all had a typical quadripartite structure: large single copy region (LSC), small single copy region (SSC), and two inverted repeat regions (IRs) (Figure 1). The length of the LSC region in M-S-I-P-M2 cp genomes is from 87,789 to 88,460 bp, the length of SSC is from 18,723 to 19,122 bp, and the length of IRb is from 25,689 to 25,711 bp (IRa: 25,691–25,713 bp) (Supplementary Table S1). All cp genomes have been uploaded to NCBI (Genebank: MT249113–MT249117).

Through gene annotation, we found that the chloroplast genome of five *Pterocarpus* species (M-S-I-P-M2) showed similar genome structures, containing 110 unique genes (77 protein coding genes, 4 rRNA, and 29 tRNA). Eighteen of the genes (atpF, matK, ndhA, ndhB, ndhI, petB, petD, rpl16, rpl2, rpoC1, rpoC2, rps12, trnA-UGC, trnI-GAU, trnK-UUU, trnL-UAA, trnV-UAC, ycf68) contain one intron and two genes (ycf3; clpP) contain two introns (Table 1). There is one intron in the ycf1 gene of *P. marsupium*, *P. pedatus,* and *P. santalinus,* but it is absent in *P. indicus* or *P. macrocarpus*. In all the cp genomes of *Pterocarpus*, the ycf1 gene spans the SSC and IRa junction. The rps12 is a trans-spliced gene similar to those found in the genera *Diospyros* and *Ziziphus* [32,33]. The 5^‘^ end is located in LSC region, and the 3^‘^ end in the IRa and IRb region. Protein-coding genes account for 49.4–49.7% of the total genome length while intergenic regions and introns account for 50.3–50.6%. In M-S-I-P-M2 cp genomes were AT-rich, with 63.61–63.69% of the genome made up of A/T nucleotides. The A/T content of protein coding genes, tRNA and rRNA among the five *Pterocarpus* cp genomes was similar, with 62.3–62.32%, 46.72%, and 44.54% A/T content respectively (Supplementary Table S1).

### 2.2. Detection of Chloroplast Repeat Sequences and SSRs

In this study, we identified 107, 136, 127, 138, and 95 repeat sequences in M-S-I-P-M2 cp genomes respectively, of which palindromic was the most common type, accounting for 37.5–42.1% of all the repeats, followed by forward (27.5–33.6%), reverse (15.8–21.3%), and complementary (9.4–14.7%). Most of the repeats in the genomes were found in the non-coding regions, with some in the coding regions accD, ndhF, ndhI, psaA, psaB, rpoC2, rps19, trnR-UCU, trnS-GCU, trnS-GGA, trnS-UGA, ycf1, and ycf2 (Supplementary Table S2).

**Figure 2:**
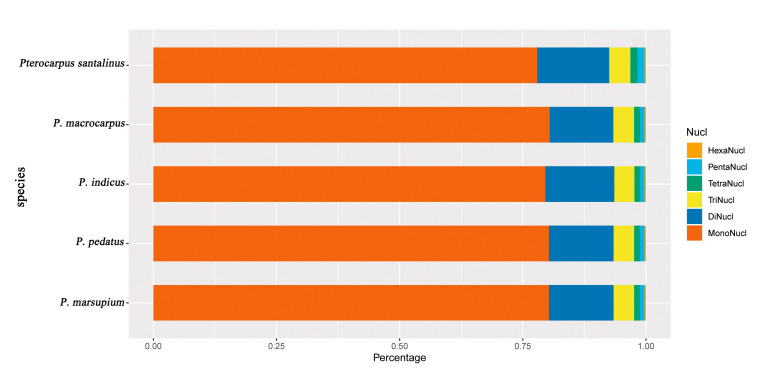
The abundance of different SSR types in the five chloroplast genomes of *Pterocarpus* (M-S-I-P-M2 cp genomes).

We detected 333, 349, 343, 335, and 335 SSR loci in M-S-I-P-M2 cp genomes, respectively. The majority of the SSRs were A/T homopolymers (proportion of A/T in SSRs: 72.8–76.4%; proportion of C/G in SSRs: 3.9–5.8%). The single nucleotide repeats motifs accounted for 77.9–80.5% of all the SSR types and of those, repeats of 7–11 accounted for 85.1–87.5% of the single nucleotide repeats. There were 43–51 dinucleotide repeats, accounting for 12.9–14.6% of the SSRs. In the di-nucleotide repeat category, AT/AT repeats were more frequent in *Pterocarpus* with 90.7–92.2% of the loci being this type. For trinucleotide repeats in *Pterocarpus*, the AAT/ ATT were observed most frequently with 92.9–93.3% of trinucleotide SSRs being of this type (AAG/CTT: 6.7–7.1%), with all trinucleotide repeats together accounting for 3.8–4% of all SSRs. The frequency of other repeat types (tetranucleotide, pentanucleotide, hexanucleotide) was very low in *Pterocarpus*, accounting for a total of 2.4–3.2% (Figure 2). In all cp genomes, the number of SSRs present in all kinds of groups was 39.6–40.7% in the total number of SSRs (Supplementary Table S3).

### 2.3. IR Expansion and Contraction

**Figure 3:**
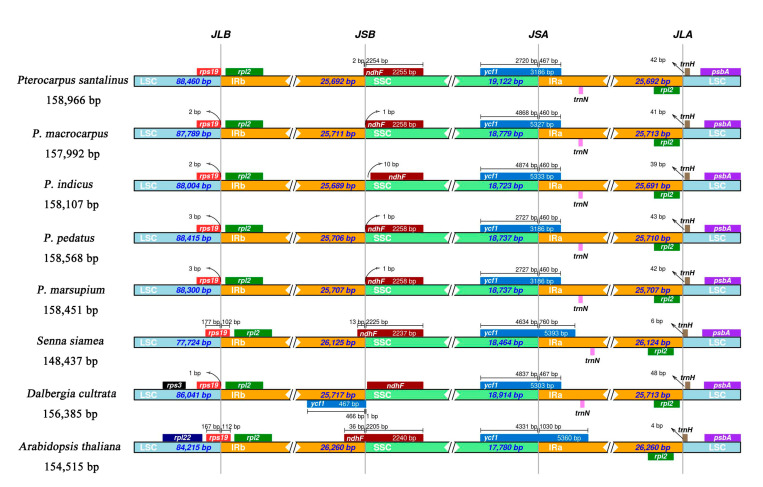
Comparison of LSC, IRb, SSC, and IRa border regions in five species of *Pterocarpus*, *Dalbergia cultrata*, and *Arabiopsis thaliana.*

We analyzed the junctions of the IRs and the two single copy regions, along with placement of adjacent genes in the M-S-I-P-M2 cp genomes and three reference cp genomes (*Senna siamea*, Mn 525772; *Dalbergia culturata*, NC_044117; *Arabidopsis thaliana*, KX 551970). The genes located at the junctions included (IRs, LSC, SSC) rps19, rpl2, ndhF, ycf1, and trnH. The rps19 and rpl2 genes were detected at the junction of LSC and IRb. The rpl2 gene is entirely located within the IR regions. The rps19 gene of all *Pterocarpus* and *D. culturata* is similar in location, and both are located in the LSC region, 0–3 bp away from the LSC and IRb boundary, which is different from the rpl2 gene in *Senna siamea* and *Arabidopsis thaliana* which span the LSC and IRb boundary. The ndhF gene of *P. macrocarpus*, *P. indicus*, *P. pedatus,* and *P. marsupium* is located in the SSC region, 1–10 bp away from the boundary of IRb-SSC. The ndhF gene of *P. santalinus*, *Senna siamea* and *Arabidopsis thaliana* is mainly located in SSC region, but spans the junction 2 bp, 13 bp, and 36 bp into the IRb region, respectively. The ycf1 gene in all genomes compared herein spans the SSC, IRa junction with the majority of the gene located in the SSC region. The length of the ycf1 gene in the IRa ranges from 460–467 bp in *Pterocarpus* and *D. culturata*. The length of ycf1 gene in the IRa of *Senna siamea* and *Arabidopsis thaliana* is 760 bp and 1030 bp respectively. The trnH gene is located at the junction of IRa and LSC, and it is totally contained in the LSC region (distance from IRa / LSC boundary: *P. santalinus*, 42 bp; *P. macrocarpus*, 41 bp; *P. indicus*, 39 bp; *P. pedatus*, 43 bp; *P. marsupium*, 42 bp; *Senna siamea*, 6 bp; *D. culturata*, 48 bp; *Arabidopsis thaliana*, 4 bp). *Pterocarpus* and *D. culturata* ycf1 genes are 39–48 bp away from IRa LSC junction, while *Senna siamea* and *Arabidopsis thaliana* are 4–6 bp away. The length of the IR region of the chloroplast genomes in M-S-I-P-M2 and *D. culturata* is similar (IRb: 25,689–25,717 bp; IRa: 25,691–25,713 bp). In terms of chloroplast genome and IRs region length, there is a sizeable difference between M-S-I-P-M2 (IRb: 25,689–25,711 bp; IRa: 25,691–25,713 bp; chloroplast genomes: 157,992–158,966 bp), *Senna siamea* (IRb: 26,125 bp; IRa:26,124 bp; chloroplast genome: 148,437 bp), and *Arabidopsis thaliana* (IRb: 26,260 bp; IRa: 26,260 bp; chloroplast genome: 154,515 bp) (Figure 3).

### 2.4. Sequence Variation of Chloroplast Genome

**Figure 4:**
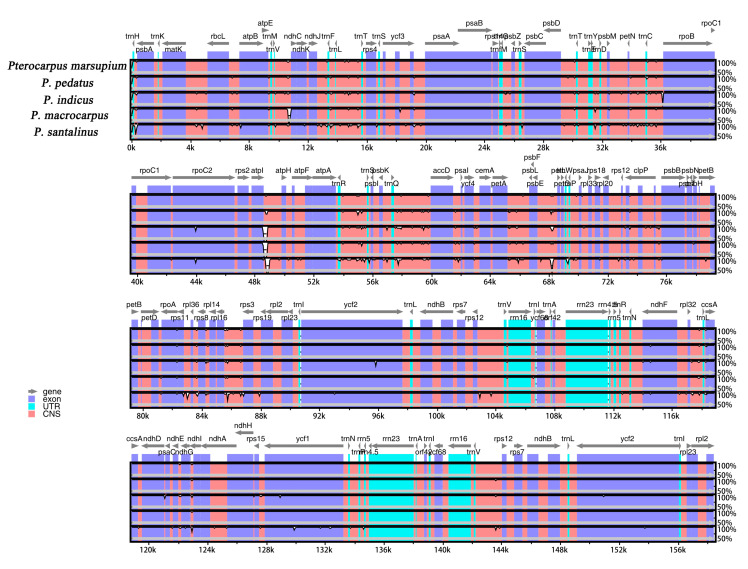
Global alignment of five (M-S-I-P-M2) chloroplast genomes of *Pterocarpus* using mVISTA. Y-axis indicates the range of identity (50–100%). Alignment was performed using *Pterocarpus marsupium* as a reference.

**Figure 5:**
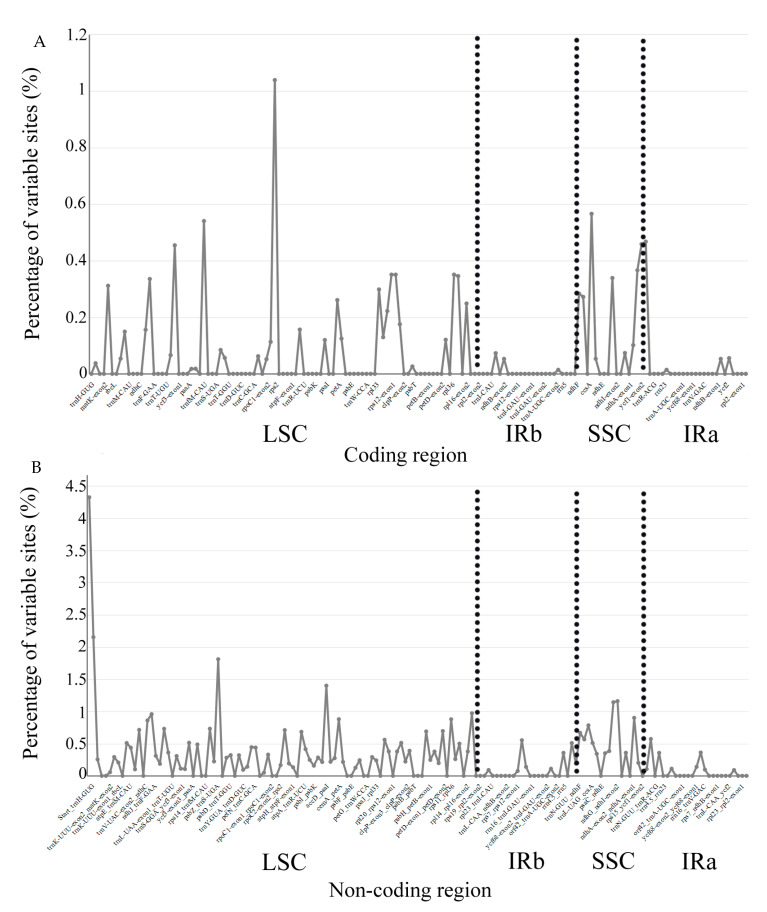
Comparison of nucleotide variability in coding region (**A**) and non-coding region (**B**) among our new sequenced five species of *Pterocarpus*.

We analyzed the sequence differences of M-S-I-P-M2 cp genomes using mVISTA. All *Pterocaprus* cp genome sequences showed very high sequence similarities. The highly variable regions were mainly concentrated in the non-coding sequences, while the exons, introns and ncRNA generally had little variation between genomes (Figure 4). In the non-coding regions, the regions with large variation are Start_trnH-GUG, trnH-GUG_psbA, trnS-UGA_psbC, accD_psaI, ndhI-exon2_ndhI-exon1, and ndhG_ndhI-exon2 (Pi > 0.01). In the coding regions, the most variable gene was rpoC2-exon2 (Pi = 0.01), followed by ccsA (Pi = 0.006) and trnfM-CAU (Pi = 0.005). The most variable regions in the genomes were located in LSC and SSC regions, with the IR regions remaining relatively conserved across the genus (Figure 5).

### 2.5. Selection on Functional Genes

In order to find patterns of selection in protein coding genes, the synonymous and non-synonymous changes were compared from the five *Pterocaprus* cp genomes using *P. santalinus* as the reference. The Ka /Ks of 77 protein coding genes in five chloroplast genomes was calculated and compared. The Ka range of M-S-I-P-M2 cp genomes was from 1.94 × 10^−6^ to 2.27 × 10^−2^, and the Ks range was from 1.76 × 10^−5^ to 6.75 × 10^−2^. The genes inferred to be undergoing positive selection were (ω > 1) accD, ndhB, ndhD, rpl32, rps4, and ycf2 (the highest ratio of Ka/Ks was for the ycf2 gene between *P. santalinus* and *P. indicus*, ω =50). Protein coding genes inferred to be undergoing purifying selection (ω < 1) across all five *Pterocaprus* cp genomes include atpB, clpP, ndhH, psaA, psbB, psbC, rpl36, and rps12 (ω = 0.001) (Table 1). 

### 2.6. Phylogenetic Analysis Based on Chloroplast Genome

**Figure 6:**
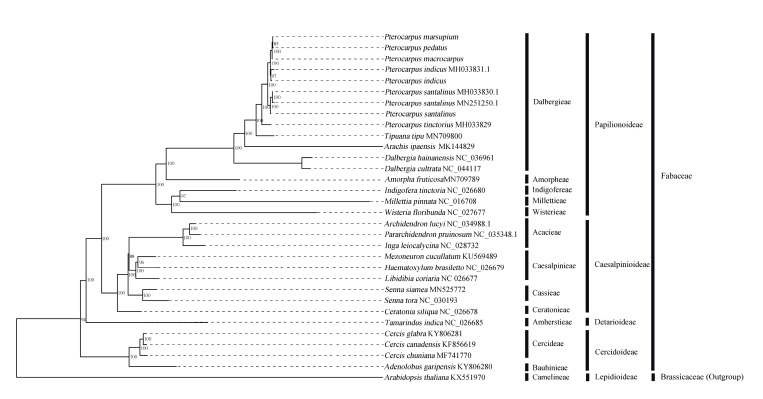
Phylogenetic tree for 32 species in Fabaceae (*Arabidopsis thaliana* as an outgroup) using maximum likelihood (ML), based on alignments of complete chloroplast genomes. Numbers at the nodes indicate bootstrap values from 1000 replicates. If the bootstrap values are as 100, this number was not shown on the nodes.

We adopted the six subfamilies proposed by the Legume Phylogeny Working Group and employed 32 cp genomes (including the five *Pterocaprus* cp genomes newly sequenced in this study) from four of these subfamilies to infer phylogenetic relationships by employing the maximum likelihood (ML) and Bayesian inference (BI) methods. Support values (BS) across the phylogenetic tree are consistently very high except for the branch leading to Acacieae and Caesalpinieae (BS = 88 for ML and 1.0 for BI, Supplementary Figure S1). Seven species (including nine individuals) of Pterocarpus (M-S-I-P-M2, *P. tinctorius* and *P. santalinus*) formed a monophyletic group with high support (BS = 100 for ML). The branch support value (BS = 85) for *P. marsupium* and *P. pedatus* was lower than the other branch support values in *Pterocaprus*. All chloroplast sequences from individuals with the same taxonomic designation resolved in the same clade with high support. However, these chloroplast sequences from the same species did not resolve in a polytomy indicated that within species differences are present in the chloroplast. Within the tribe Dalbergieae, *Pterocarpus* was resolved as sister to *Tipuana tipu*. Further, *Dalbergia* was resolved as an early diverging lineage in this clade (BS = 100, Figure 6). Increased samplings of species within this group could be added to the current dataset to correctly infer the relationships of unresolved taxa. In addition, we calculated the divergence times among the 32 sampled individuals and found that the chloroplast genome dataset provided similar divergence times as those from other genetic datasets (Supplementary Figure S2) [34].

## 3. Discussion

From the whole cp genomes sequenced thus far in plants, a length 120–160 kb has been found [35,36]. In this study, we sequenced the cp genomes of M-S-I-P-M2, with a length of 157,992–158,966 bp. Therefore, *Pterocarpus* cp genomes are among some of the larger cp genomes sequenced thus far, even when compared to other species in Fabaceae (Figure 3, *Senna siamea* 148,437 bp). The expansion of cp genomes is often correlated with expansion and contraction IR/SC junctions [37] which is evident in the comparisons of *Pterocaprus* species to *Senna siamea* and *Arabidopsis thaliana* (Figure 3). Like most angiosperms, the five *Pterocarpus* cp genomes are similar in structure and consistent to other plant cp genomes with 110 genes [38,39,40] and a high A /T content, which was found to be as high as 63% [33,41,42]. The lower G/C content may be related to the spontaneous mutations in genomes of *Pterocarpus* [43].

SSR markers have high mutation rates and can be used as a molecular marker for population genetics, phylogenetic inference, and biogeographic studies [44,45,46]. In this study, we detected 333–349 SSR loci in M-S-I-P-M2 cp genomes. The proportion of single nucleotide repeats of A/T is the highest among all the repeat types (72.8–76.4%). This suggests that cp genome contains not only abundant A /T content, but also a large number of short polyadenine (PolyA) /polythymine (poly T) repeats which are associated with polyadenylation at the terminus of mRNA genes [33]. The mono-nucleotide repeats were the most abundant repeats and accounted for 77.9–80.5%. In di-nucleotide repeats, the AT/TA motif was most common. In tri-nucleotide repeats, the AAT/ ATT motif was most common. The SSR loci containing A and T are more common in *Pterocarpus*. With the increase in motif length, the frequency of SSRs decreased. These are similar to the structural characteristics of chloroplast SSR in dicots. SSR polymorphism is a repeat length polymorphism caused by the elongation or shortening of repeat units [47]. It is widely used for population genetic diversity or population classification analysis [46,48]. At present, the molecular mechanism for the origin of microsatellites is not completely clear. Replication slippage, unequal crossing over, and nucleotide substitution are all possible mechanisms for creating microsatellite variations but do not explain the origin of SSRs [49,50,51]. The SSR marker is one of the common molecular means to study the evolution of species [52,53]. SSR markers can be easily genotyped and often can be used as molecular markers in many related species [54,55,56]. Therefore, SSRs identified from the chloroplast genomes in this study could be valuable markers for future studies in ecology, evolution and tracking of timber.

The IR region is highly conserved and is thought to play an important role in stabilizing the structure of cp genomes [57]. The contraction and expansion of IR regions is a common phenomenon in cp genome evolution, which can contribute to overall length variation of cp genomes [33]. By comparison, we found that the length of M-S-I-P-M2 cp genomes and IR regions were tightly associated in length variation (changes in the length of chloroplast genome ≤ 974 bp; changes in the length of IR region ≤ 22 bp). Compared with *Pterocarpus*, *Arabidopsis thaliana* and *Senna siamea* have longer IR regions and smaller genomes. This has resulted in differences of gene placement at the IR/SC boundaries (rps19, ndhF cross LSC / IRb and IRb / SSC boundaries respectively; trnH distance from IRa / SSC boundary ≤ 6 bp) between them and M-S-I-P-M2 cp genomes (Figure 3) [58]. Our results show that the cp genomes of *Pterocarpus* may be conserved in gene content in comparison to other angiosperms but have expanded in overall genome length suggesting ongoing evolution in *Pterocarpus* cp genomes [33,59]. All genes are similarly located across all five species in *Pterocarpus* (and *Dalbergia*) in respect to the IR/SC junctions, suggesting that expansion and contraction in the IR and SC regions has not resulted in large changes to the junction boundaries in Dalbergieae.

Because of the highly conserved structure and nucleotide content of plant cp genomes mutational hotspots can be easily identified using comparative analyses. These mutational hotspots flanked by conserved sequences are the basis for highly variable markers (DNA barcodes) often used in population genetic or phylogenetic research [60,61]. In this study, we used mVISTA to compare the whole cp genome sequences of five *Pterocarpus* species and calculated the percentages of variable characters in the coding region and non-coding regions in order to identify such variable regions. We found that, similar to previous plant studies, the non-coding regions were more variable than the coding regions [62,63,64]. Similar to *Artemisia annua* and *Panax notoginseng*, the variation of the SC regions in *Pterocarpus* cp genomes is greater than that in IR regions [65,66]. In the analysis of chloroplast genome sequence variation, we detected nine highly variable regions in coding (rpoC2-exon2, ccsA, and trnfM-CAU) and non-coding regions (Start_ trnH-GUG, trnH-GUG_psbA, trnS-UGA_psbC, accD-psaI, ndhI-exon2_ndhI-exon1, and ndhG_ndhi-exon2). In previous studies, in addition to the Start_ trnH-GUG and ndhI-exon2_ndhI-exon1 regions, seven highly variable regions have been used as DNA barcodes in other plants or are in the process of being developed as DNA barcodes [67,68,69,70,71]. Our results showed that the Pi of ndhI-exon2_ndhI-exon1 in *Pterocarpus* is more than 0.01, which is considered a highly variable region. We suggest that trnH-GUG_psbA, trnS-UGA_psbC, accD-psaI, ndhI-exon2_ndhI-exon1, ndhG_ndhi-exon2, rpoC2-exon2, ccsA, and trnfM-CAU, the regions with the highest degree of variation in the chloroplast genome of *Pterocarpus*, be used as DNA barcodes. These highly variable regions may also be useful for the resolution of interspecific relationships of *Pterocarpus* in the phylogeny of legumes.

Non-synonymous (Ka) and synonymous (Ks) mutations, and the ratio between these (ω = Ka/Ks) when compared across different species or populations, can be used to infer what type of selection is acting upon different genes [72]. Because the cp genome is a non-recombining genome [73], it is rare to have a large number of recombination events, resulting in a low DNA replacement rate and gene conservation [62,74,75,76]. Despite the lack of recombination, purifying selection is still an important mechanism in cp genomes for maintaining a given gene function through time [77]. In this study, we found that most of the genes of *P. santalinus*, compared with those of *P. macrocarpus*, *P. indicus*, *P. pedatus* and *P. marsupium*, has ω < 1 suggesting that purifying selection has been important in maintaining conserved gene residues. The regions with high purifying selection pressure were mainly found among the genes related to photosynthesis (Subunits of photosystem II and Subunits of ATP synthase; Table 1). Similar to the evolution of WRKY family in Gramineae, strong purifying selection conserves specific gene residues and gene functions across species in *Pterocarpus* [78]. Other genes of *Pterocarpus* were inferred to be under strong positive selection and were mainly found in genes for self-replication (large subunits of ribosome and small subunits of ribosome), photosynthesis (subunits of NADH dehydrogenase), and genes of unknown function (ycf genes) (Table 1). As such, positive selection may promote the functional divergence between these genes [79].

In this study, the M-S-I-P-M2 cp genomes were used to analyze the phylogenetic relationship of *Pterocarpus* and the phylogenetic position of *Pterocarpus* in Fabaceae. The phylogenetic analysis shows that Pterocarpus is a monophyletic group [80]. Among the seven cp genomes of *Pterocarpus* (M-S-I-P-M2, *P. tinctorius,* and *P. santalinus*), *P. tinctorius* was resolved as early diverging [4]. The results from our phylogenetic analyses concur with those from other studies demonstrating the utility of cp phylogenomic in plant systematics studies of Fabaceae [28,81,82]. In addition, the branching pattern among samples from the same species of *Pterocarpus* indicate that the chloroplast data could be used for studying intraspecific relationships in this genus.

## 4. Materials and Methods

### 4.1. Plant Material

We collected fresh leaves from five species of *Pterocarpus* for DNA extraction. The leaf material of *P. macrocarpus* was collected in Baan Loom Soom, Saiyok, Kanchanaburi, Thailand (14.22° N, 99.20° E). The leaf material of *P. santalinus* was collected from Calcutta, West Bengal, India (23.35° N, 88.52° E) and the *P. indicus* leaf material was collected from Hoskote, Bangalore, Karnataka, India (13.1° N, 77.8° E). The leaf material of *P. pedatus* and *P. marsupium* were collected at the Experimental Station of Research Institute of Tropical Forestry, Chinese Academy of Forestry, Jianfeng Town, Ledong Li Autonomous County, China (18.69° N, 108.79° E). 

### 4.2. DNA Extraction and Sequencing

We extracted high-quality DNA from fresh leaves using an E.Z.N.A^®^ Plant DNA kit (Omega Bio-Tek Inc., Norcross, GA, USA). The DNA quality was assessed with an Agilent 2100 Bioanalyzer (UC Davis Genome Center, Davis, CA, USA) [83]. The total DNA was sequenced on an Illumina HiSeq X Ten platform, a Hiseq 4000 (Illumina Inc., San Diego, CA, USA) and using long read sequencing on a Pacific Biosciences (Shanghai, China; PacBio; Preparing Arabidopsis Genomic DNA for Size-Selected~ 20 kb SMRTbell™ Libraries) Sequel platform at the same time. For Illumina sequencing, libraries with the paired-end short-insert of 450 bp were generated for the HiSeq X Ten platform. The, the DNA libraries were also sequenced on Illumina Hiseq 4000 [84]. For PacBio sequencing, 20 kb libraries were generated and sequenced on the PacBio Sequel instrument [85].

### 4.3. Sequence Assembly of cp Genomes

The raw sequencing data from the Illumina reads was first filtered and quality controlled through Trimmomatic v0.39 (available online: http://www.usadellab.org/cms/?page=trimmomatic) to remove the reads of low quality (>10% Ns, > 40% low quality bases or small segments with length less than 75 bp after pruning). The clean reads were initially assembled with ABySS v2.0.2 (available online: http://www.bcgsc.ca/platform/bioinfo/software/abyss). The long read PacBio data were corrected by mapping the high depth Illumina reads onto the PacBio scaffolds [86]. Assembly of chloroplast genome (illumina data+ PacBio data) was done using SPAdes-3.13.0 (available online: http://cab.spbu.ru/software/spades/) [87]. The assembled sequences were then checked against the Nucleotide Sequence Database (GeneBank; available online: https://www.ncbi.nlm.nih.gov/) to confirm the chloroplast scaffold sequences. The chloroplast assembly results were optimized by aligning with the original clean Illumina reads using the Burrows-Wheeler Aligner (available online: http://bio-bwa.sourceforge.net/) and base correction is then performed with Pilon v1.22 (available online: https://github.com/broadinstitute/pilon). [88].

### 4.4. Gene Annotation

The reference genome (*Saccharum hildebrandtii*, GenBank: MF563371.1) was used to correct the starting position of the assembled chloroplast genomes and determine the position and direction of the chloroplast junction boundaries (LSC / IRa / SSC / IRb). The genes of the chloroplasts were annotated using DOGMA via the online server [89]. To remove the redundancy of the predicted genes, the *Saccharum hildebrandii* chloroplast genome sequence (GenBank: MF563371.1) was used as a reference to manually correct the head and tail of the gene and exon / intron boundaries. The gene map of the cp genomes was drawn using OrganellarGenomeDRAW v1.3.1 (available online: https://chlorobox.mpimp-golm.mpg.de/OGDraw.html) [90]. BLAST searches (E-value <= 1E-5, minimal alignment length percentage >= 40%) for whole chloroplast genome were performed against 5 databases. Kyoto Encyclopedia of Genes and Genomes [91,92,93,94], Clusters of Orthologous Groups [95], Non-Redundant Protein Database databases [96], Swiss-Prot [97], and Gene Ontology [98] were used to properly annotate the functional information of each coding gene.

### 4.5. Identification of Repeat Sequences and Simple Sequence Repeats (SSR)

Repeat sequences in the cp genomes were identified using the program REPuter (available online: http://bibiserv.techfak.uni-bielefeld.de/reputer/) and included four types of repeat: forward repeat (F), reverse repeat (R), complementary repeat (C) and palindromic repeat (P). Detection parameter settings were as minimum repeat size 30 bp, and an edit distance of 3.

SSR identification was carried out on the cp genome sequences by MIcroSAtellite identification tool (MISA; available online: http://pgrc.ipk-gatersleben.de/misa/http://pgrc.ipk-gatersleben.de/misa/) with the parameter settings: unit-size (nucleotide) _min-repeats: 1_8, 2_5, 3_4, 4_3, 5_3, 6_3. The minimum distance between two SSRs was set to 100 bp [99].

### 4.6. Polymorphism Analysis, Comparison of Genome Structure, and IR Region Contraction and Expansion Analysis of M-S-I-P-M2 cp Genomes

The divergence among different cp genomes and the identification of mutational hot spots was done by quantifying nucleotide variability in DnaSP v5.10 on coding and non-coding sequences separately [100]. The multiple sequence alignment for cp genome was conducted using MAFFT v.7 [101]. The expansion and contraction of the IR regions will change the length and structure of cp genome overall, which can change the copy number of adjacent genes and lead to the formation of pseudogenes [102]. Eight cp genomes (M-S-I-P-M2; *Dalbergia cultrata*, NC_044117; *Senna siamea*, MN 525772; and *Arabidopsis thaliana*, KX 551970) were selected to compare the location of IRs, the SSC and LSC junctions in different genomes. The boundary differences among the five *Pterocarpus* cp genomes (M-S-I-P-M2) were visualized using mVISTA (default parameters and Shuffle-LAGAN mode) [103].

### 4.7. Gene Selective Pressure Analysis

In order to assess the selection pressure of genes in the cp genomes of *Pterocarpus*, we calculated the synonymous (Ks) and non-synonymous (Ka) mutation rates of exons of orthologous coding genes (77) in the M-S-I-P-M2 cp genomes (*P. santalinus* was used as a reference) using Geneious v9.0.5 (Biomatters, Auckland, New Zealand). The analysis of selection pressure is calculated by the ratio of non-synonymous (Ka) to the synonymous mutation rate (Ks) (ω = Ka / Ks). We also combined all the coding gene as one huge data matrix to evaluate the selection for this *Pterocarpus* clade by using the PAML method.

### 4.8. Phylogenetic Analysis of Chloroplast Genomes

In this study, in addition to the five new sequenced M-S-I-P-M2 cp genomes of *Pterocarpus*, an additional 27 cp genomes (from 2 families, 5 subfamilies and 12 tribes, outgroup: *Arabidopsis thaliana*, Supplementary Table S4) were downloaded from NCBI to resolve a chloroplast phylogenetic tree. The sequences were aligned using ClustalW (v2.0.12) with the default settings. The DNA substitution model chose was assessed using the Akaike information criterion (AIC) method [104]. Maximum likelihood (ML) phylogenetic inference was done using RAxMLv8.2.6 with 1000 bootstrap pseudo-replicates to assess branch support [105,106]. Bayesian inference (BI) using MrBayes3.1.2 was also employed based on the method from Wu et al. [107].

## 5. Conclusions

In this study, the complete cp genomes of five species of *Pterocarpus* (*P. macrocarpus*, *P. santalinus*, *P. indicus*, *P. pedatus* and *P. marsupium*) were sequenced by high-throughput sequencing for the first time. Through comparison, we found that the cp genomes of five species have similar structural characteristics and have the typical four-part structure as other land plants. The chloroplast genome contains abundant SSR loci (A/T as the main component). Through nucleotide variation analysis, we found that eight mutational hot spots (trnH-GUG_psbA, trnS-UGA_psbC, accD-psaI, ndhI-exon2_ndhI-exon1, ndhG_ndhi-exon2, rpoC2-exon2, ccsA and trnfM-CAU) could be used as DNA barcode regions for *Pterocarpus*. In the comparison of gene selection pressures (*P. santalinus* as the reference genome), purifying selection was found to be important in maintaining conserved gene function. Phylogenetic analysis shows that *Pterocarpus* is a monophyletic group. The finished cp genomes of *Pterocarpus* and comparative analyses provide numerous different types of genetic markers with uses ranging from population genetic studies to the tracking of the origin of timber.

## Figures and Tables

**Table 1:** List of genes encoded by five species of Pterocarpus chloroplast genome. (×2) indicates that the gene has two copies. * and ** indicate genes containing one/ two introns. The ycf1 gene of *Pterocarpus pedatus*, *Pterocarpus marsupium* and *Pterocarpus santalinus* contains one intron. The cp genome of *P. santalinus* was used as the reference, + indicates that the gene is inferred to be under positive selection (ω > 1) and − indicates that the gene is inferred to be under purifying selection (ω = 0.001).

Category	Group	Genes
Photosynthesis	Subunits_of_photosystem_I	psaA^-^, psaB, psaI, psaJ, psaC
	Subunits_of_photosystem_II	psbA, psbZ, psbC^-^, psbD, psbM, psbI, psbK, psbL, psbF, psbE, psbB^-^, psbT, psbN, psbH
	Subunits_of_NADH_dehydrogenase	ndhC, ndhK, ndhJ, ndhB(×2) * ^+^, ndhF, ndhD^+^, ndhE, ndhG, ndhI *, ndhA *, ndhH^-^
	Subunits_of_cytochrome_b/f_complex	petN, petA, petL, petG, petB *, petD *
	Subunits_of_ATP_synthase	atpB^-^, atpE, atpI, atpH, atpF *, atpA
	Large_subunit_of_Rubisco	rbcL
Self-replication	Large_subunits_of_ribosome	rpl33, rpl20, rpl36^-^, rpl14, rpl16 *, rpl2(×2) *, rpl32^+^, rpl23(×2)
	Small_subunits_of_ribosome	rps4^+^, rps14, rps2, rps18, rps11, rps8, rps3, rps19, rps12 *^-^ (×2,part), rps15, rps7(×2)
	DNA-dependent_RNA_polymerase	rpoB, rpoC1 *, rpoC2 *, rpoA
	Ribosomal_RNAs	rrn5, rrn4.5, rrn23, rrn16
	Transfer_RNAs	trnH-GUG, trnK-UUU *, trnM-CAU, trnV-UAC *, trnF-GAA, trnL-UAA *, trnT-UGU, trnS-GGA, trnfM-CAU, trnG-UCC, trnS-UGA, trnT-GGU, trnE-UUC, trnY-GUA, trnD-GUC, trnC-GCA, trnR-UCU, trnS-GCU, trnQ-UUG, trnW-CCA, trnP-UGG, trnI-CAU, trnL-CAA, trnV-GAC, trnI-GAU *, trnA-UGC *, trnR-ACG, trnN-GUU, trnL-UAG
Other genes	Maturase	matK *
	Protease	clpP **^-^
	Envelope_membrane_protein	cemA
	Acetyl-CoA_carboxylase	accD^+^
	C-type_cytochrome_synthesis_gene	ccsA
Genes of unknown function	Proteins_of_unknown_function	ycf3 **, ycf4, ycf68(×2) *, ycf1, orf42(×2), ycf2(×2) ^+^

## Supplementary Materials

Supplementary materials can be found at https://www.mdpi.com/1422-0067/21/11/3758/s1. Table S1. The M-S-I-P-M2 cp genomes of Pterocarpus assembly, size, and features. Table S2. The repeats (≥30 bp) identified in M-S-I-P-M2 cp genomes (repetition type; F: Forward, R: Reverse, C: Complement, P: Palindromic). Table S3. The SSR positions in M-S-I-P-M2 cp genomes. Table S4. The information of 22 chloroplast genomes downloaded from Genebank (NCBI, https://www.ncbi.nlm.nih.gov/). Figure S1. Phylogenetic tree for 32 species in Fabaceae (*Arabidopsis thaliana* as an outgroup) using Bayesian inference (BI), based on alignments of complete chloroplast genomes.; Figure S2. Divergence times among the 32 sampled individuals based on Bayesian inference.
