# Supplementary figures and images for: Comparative Analyses of Five Complete Chloroplast Genomes from the Genus Pterocarpus (Fabacaeae)
Source: Int J Mol Sci. 2020 May 26;21(11):3758. doi: 10.3390/ijms21113758 (PMC7312355; doi:10.3390/ijms21113758)

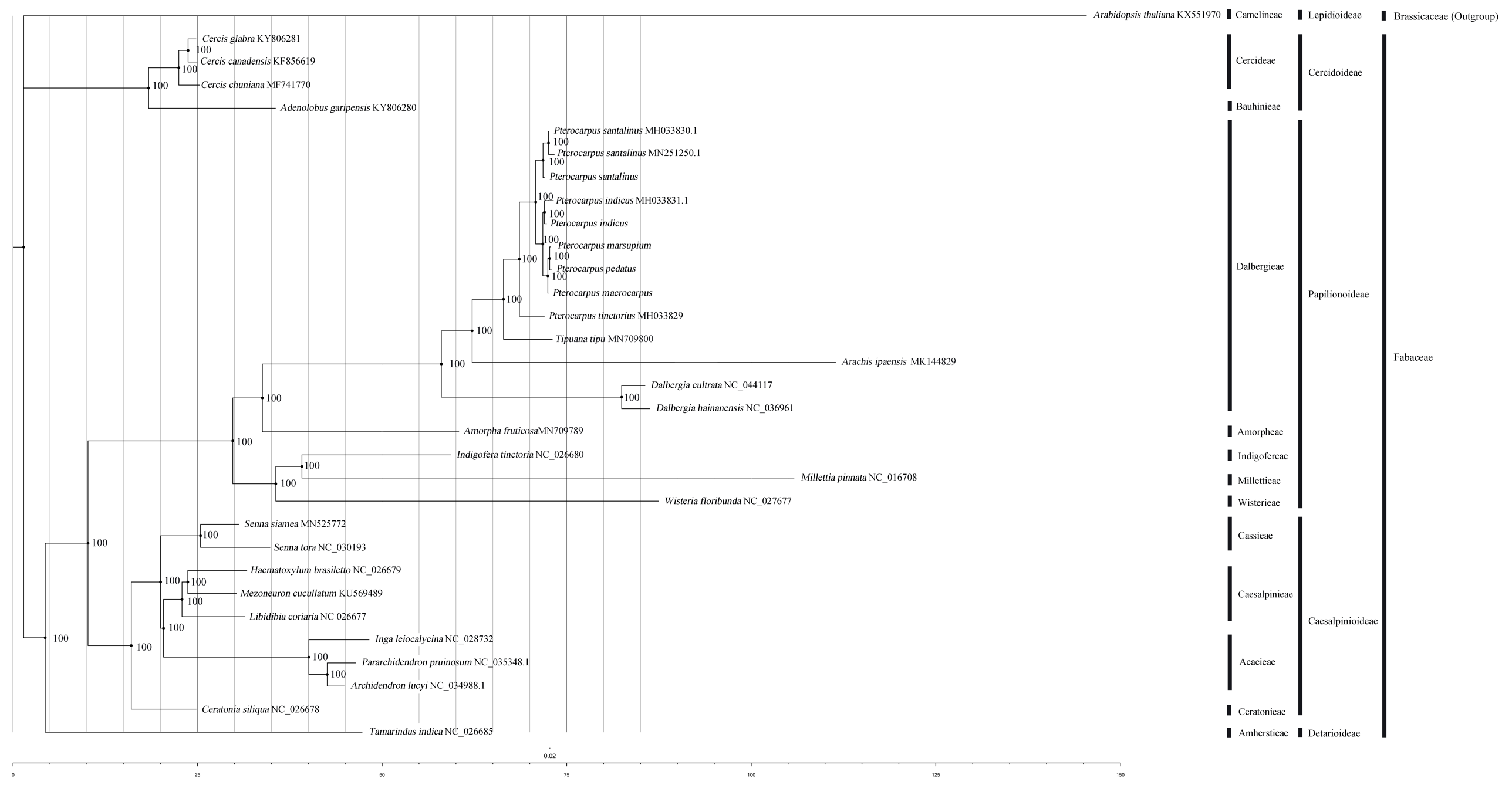

Supplement: Supplementary file 1 [file ijms-21-03758-s001.zip › Supp Fig 2-BI-timetree.jpg]

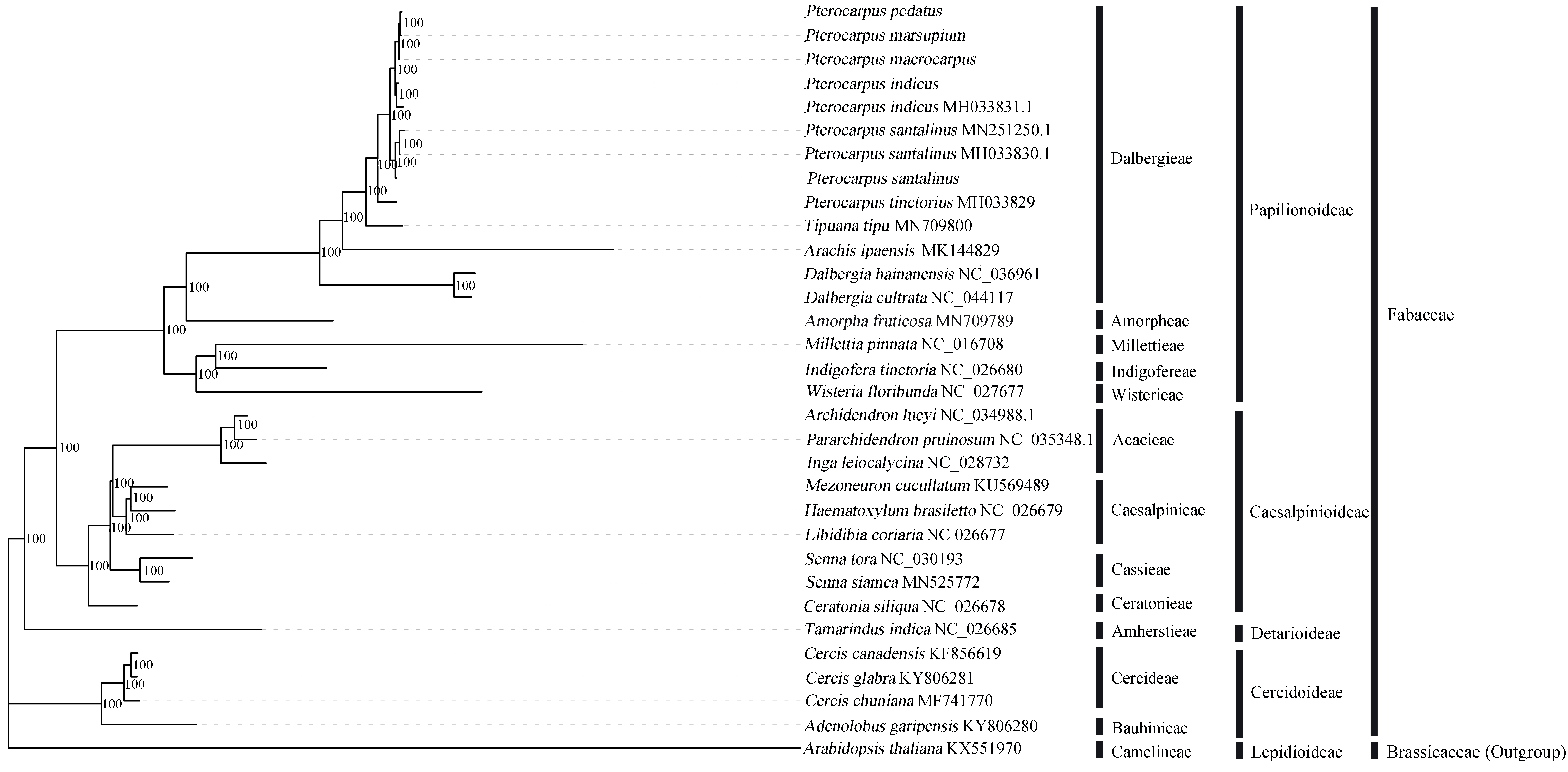

Supplement: Supplementary file 1 [file ijms-21-03758-s001.zip › Supp Fig 1-BI-tree.jpg]
